# How can community participation strengthen a health insurance system? The case of health insurer’s user associations in Colombia

**DOI:** 10.1136/bmjgh-2022-009571

**Published:** 2022-09-16

**Authors:** Mery Bolivar-Vargas, Eduardo Alfonso-Sierra, Janet Bonilla, Martha Herrera, Haydee Rodriguez, Manuela Villar-Uribe, Kerry Scott, Inke Mathauer

**Affiliations:** 1 Faculty of Economic and Administrative Sciences, Universidad Jorge Tadeo Lozano, Bogotá, Colombia; 2 Centre for Medicine and Society, Albert-Ludwigs-University Freiburg, Freiburg, Germany; 3 Health Nutrition and Population Global Practice, World Bank Group, Bogotá, Colombia; 4 Executive Director, Colombian Foundation for Orphan Diseases, Bogotá, Colombia; 5 Health Nutrition and Population Global Practice, World Bank Group, Washington, District of Columbia, USA; 6 Department of International Health, Johns Hopkins University Bloomberg School of Public Health, Baltimore, Maryland, USA; 7 HGF, World Hlth Org, Geneva, Switzerland

**Keywords:** Health insurance, Health policy, Health systems, Public Health

## Abstract

**Background:**

Colombia’s universal health coverage programme has enrolled 98% of the population, thereby improving financial protection and health outcomes. The right to participate in the organisation of healthcare is enshrined in the 1991 Colombian Constitution. One participatory mechanism is the legal and regulatory provision that citizens can form user associations. This study examines the functionality of health insurance user associations and their influence on citizen empowerment and health insurance responsiveness.

**Methods:**

The mixed methods study includes document review (n=72), a survey of beneficiaries (n=1311), a survey of user associations members (n=27), as well as interviews (n=19), focus group discussions (n=6) and stakeholder consultations (n=6) with user association members, government officials, and representatives from insurers, the pharmaceutical industry, and patient associations. Analysis used a content–process–context framework to understand how user associations are designed to work according to policy content, how they actually work in terms of coverage, public awareness, membership, and effectiveness, and contextual influences.

**Findings:**

Colombia’s user associations have a mandate to represent citizens’ interests, enable participation in insurer decision-making, ‘defend users’ and oversee quality services. Insurers are mandated to ensure their enrollees create user associations, but are not required to provide resources to support their work. Thus, we found that user associations had been formed throughout the country, but the public was widely unaware of their existence. Many associations were weak, passive or entirely inactive. Limited market competition and toothless policies about user associations made insurers indifferent to community involvement.

**Conclusion:**

Currently, the initiative suffers from low awareness and low participation levels that can hardly lead to empowered enrollees and more responsive health insurance programmes. Yet, most stakeholders value the space to participate and still see potential in the initiative. This warrants a range of policy recommendations to strengthen user associations and truly enable them to effect change.

**WHAT IS ALREADY KNOWN ON THIS TOPIC**

- Little was known about how user associations of health insurance companies work.
- Despite almost three decades having passed since the initiative was created, only a few studies have been conducted that revealed some barriers to participate in the user associations.
- But knowledge about user association’s functionality and effectiveness was missing.

**WHAT THIS STUDY ADDS**

- This study contributes to filling the gap in knowledge by describing how user associations currently function, the extent to which they influence health insurance companies and the health system in general, and by identifying key content, process and context factors that help to explain the functionality and effectiveness of the initiative.
- The results of the study indicate there are many issues that severely limit the effectiveness of these user associations.
- Yet, most stakeholders appreciate the initiative and think user associations have potential that could be unleashed by tackling current barriers.

**HOW THIS STUDY MIGHT AFFECT RESEARCH, PRACTICE OR POLICY**

- The results of the study might promote further research on community participation in the health sector, a topic relatively neglected by researchers in Colombia, but deemed very important by the stakeholders of the system.
- Furthermore, the results could influence policy by pinpointing key factors limiting the functioning and effectiveness of health insurance user associations that could be addressed through public policy.

## Introduction

Colombia enacted a new Constitution in 1991 triggering a wide range of sociopolitical changes, including a major reform of the health system. This reform introduced a health insurance programme, which has succeeded in enroling 98% of the population as of 2021.^1^ The new Constitution also enshrined the right to participate in the organisation of the health system. This right has been operationalised in relation to Colombia’s health insurance system as a legal and regulatory provision that citizens can form health insurance user associations to represent their interests to health insurance companies. User associations are expected to receive beneficiary complaints about healthcare provision and their health insurance company as well as advise health insurance companies on how to improve their services. By doing so, these associations are envisioned as an accountability mechanism to empower citizens and make the health insurance schemes responsive to their needs.

However, in Colombia as well as in many other countries, challenges have been identified in translating formal health insurance coverage into financial protection and barrier-free utilisation of health services. The extent to which citizen participation through bodies such as user associations can reduce these barriers is of great interest. Although Colombia’s user associations have been in existence for over 25 years, little is known about their functionality and effectiveness as mechanisms for citizen empowerment and health insurance responsiveness. This paper explores the extent to which Colombia’s user associations have contributed to enabling citizens to access their entitlements and increase the responsiveness of health insurance schemes.

### Study framework

This study is part of the research programme ‘Making Health Insurance Programmes Responsive to Citizens’, supported by the Alliance for Health Policy and Systems Research, WHO. We followed an overarching framework developed for the research programme that uses Molyneux’s framework^2^ to assess how content, process and context factors, and their interactions, influence whether an accountability initiative is able to increase citizen empowerment and the responsiveness of health insurance programmes. To analyse the functionality and effectiveness of Colombia’s user associations as a health insurance accountability initiative, the framework addresses three domains: (1) content: what is the design of the user association approach and how is it supposed to be implemented? (2) Process: how does the user association actually work? (3) Context: what are the wider contextual factors at the health system, national and community levels that might influence how these user associations work?

### Colombia’s health insurance system

Health insurance in Colombia is administered by multiple health insurance companies called *Entidades Promotoras de Salud* (EPS, meaning Health Promotion Entities in Spanish). While most EPSs are shareholder-based private for profit companies, a few large public (government owned) and mixed (includes government shareholders) EPSs insure 18% of the population. In Colombia, there are two major insurance schemes. About half of all citizens are enrolled in ‘contributory scheme’ and the other half in ‘subsidised scheme’. The contributory scheme is for people working in the formal sector and their spouses and dependents, who contribute with a payroll tax of 12.5% or equivalent. The subsidised scheme is for people without ability to pay (identified through a proxy means test) and it is financed by general government revenues and a cross-subsidy of 1.5% percentage points from the payroll taxes collected for the contributory scheme.

All EPSs must provide the same benefit package, which is identical for both the contributory and subsidised schemes. It includes primary care, hospitalisation, surgery, other procedures, drugs and catastrophic diseases. EPSs have the responsibility to ‘organize and guarantee, directly or indirectly, the access to and provision of the benefit package to affiliates’ and ‘to establish procedures to ensure efficient, timely and quality care in the services provided by healthcare providers’ (Art. 178, Law 100, 1993). They exercise this responsibility partly by configuring the network of providers using a mix of public and private sector clinics and hospitals.

Colombia is divided into 32 departments and 1117 municipalities. Few health insurance companies operate country-wide, most operate regionally in several departments (and the municipalities within). The various insurance companies compete for members in the market, who are free to choose their insurer. Insurers cannot compete on contribution rates or benefits, because these are fixed, and the same services are covered. Thus, insurers are expected to attract enrollees based on quality of their interactions with members, their network of providers, waiting times for care and hospital ward amenities (eg, private rooms), among other factors, several of which are captured by the official ranking of EPSs published by the Ministry of Health. In practice, however, competition seems to be limited and markets tend to be concentrated.^3 4^

Access and utilisation have improved since the 1993 reform: for example, between 1997 and 2019, the percentage of people visiting a physician for prevention increased from 59% to 78% for the contributory scheme and from 35% to 65% for the subsidised scheme.^5 6^ Inequalities have reduced for most indicators, including morbidity and access. For example, the percentage of people having access to all medicines in a prescription increased from 25% in 2003 to 67% in 2013 for the lowest income quintile, and from 53% to 63% for the highest quintile. Nonetheless, there are persistent inequities in access, healthcare utilisation and quality of care, disproportionately affecting rural areas and the lowest wealth quintiles.^7^ Likewise, catastrophic health expenditure remains an issue. In 2016, 8% of households experienced catastrophic health expenditure at the threshold of spending 10% or more of their total household consumption on healthcare. While problematic, this value is lower than regional estimates for upper-middle income countries (16.7%) and comparable with the regional estimates for Latin America (8.7%).^8^

Continued barriers to access have resulted in widespread and persistent judicial litigation in health; lawsuits are a last-resort mechanism used by enrollees.^9^ In 2020, enrollees filed more than 80 000 lawsuits to request healthcare services with 89% of these related to services already included in the benefit package and that should have been provided.^10^ Lawsuits have proven to be an effective mechanism to get access to healthcare when it has been unjustly denied: historically, the vast majority of lawsuits have been fully or partially won by litigants; for example, in 2020, only 9% of lawsuits were rejected.

## Methods

### Study design

We followed a mixed methods approach using a sequential explanatory design^11^ drawing from document review, quantitative surveys, qualitative interviews and focus group discussions, secondary analysis of existing databases and policy discussions (table 1). We use qualitative data from interviews and focus groups primarily to explain the findings from quantitative data (survey and secondary data) that measure people’s knowledge use and overall participation in the initiative. All data sources contributed to understanding the content, process and context of Colombia’s user associations initiative.

**Table 1:** Research methods used, sampling and data collection procedures

Method	Sample and sampling	Data collection
**Desk review of key textual sources** to analyse official published documents about user association mandate and functionality.	Documents (n=72) including legal codes, regulatory and judicial decisions (25), content from insurance companies’ web sites (40), and government background documents (7). Identified through internet searches and consultation with key stakeholders.	Documents were read by authors (EA-S, MB-V, JB). Relevant content on user association mandate, envisioned mechanisms of action and actual functionality was summarised in a data extraction spreadsheet (MS Excel).
**Insurance beneficiary survey** to assess beneficiaries’ knowledge and use of user associations, as well as their health and insurance status and their overall participation and empowerment (see online supplemental annex 1)	A nationally representative sample of beneficiaries 18 and older (n=1311), in the contributory scheme (n=666) and subsidised scheme (n=645) Probabilistic sampling, stratified in two stages. Statistical strata were formed for six geographical regions and two types of municipalities (considering their weight in total population size, municipalities were segmented in two types; capitals and the rest of municipalities). In the first sampling stage, municipalities were randomly selected within each stratum and in the second stage, telephone numbers from the sampling frame were randomly selected, surveying one adult person of the selected household. The sample for the first stage comprised 50 municipalities, including 5 capitals (Bogotá, Medellín, Cali, Barranquilla, Bucaramanga). To account for the two-stage sampling design, expansion factors were calculated based on the inverse of the inclusion probabilities in the sample of the final sampling units, to obtain unbiased estimates for the parameters of interest.	The 53-item questionnaire was administered via telephone by a local firm specialised in surveys and data collection. The interviews started by describing the study and asking for consent to participate. Then filters were applied (≥18 years old, enrolled in either contributory or subsidised scheme) and the full questionnaire applied to eligible individuals. The questionnaire was programmed in a specialised software that administers the flow of the questionnaire. The interviewer entered the responses in the system. The interviews were also recorded for quality control and to codify open questions.
**User association member survey** to collect data on members’ characteristics and opinions about their user association	A non-representative sample (n=27) of user association members found through emailing a description of the study and link to the survey on user association member listservs.	The 22-item questionnaire was self-administered online and included questions on the demographic characteristics of user association members, geographical location, occupation, their role and the functioning of the user associations.
**Secondary data analysis** to assess the number and distribution of user associations	Quantitative analysis of a directory of user associations and databases of insurance beneficiaries for each insurer.	Data were requested to Superintendencia Nacional de Salud (SNS, National Health Oversight Agency) and then redirected to the publicly available data in the SNS’s web page. The directory of user associations was last retrieved in January 2021, and includes the name of the association, the municipality where it is located, the EPS to which it is linked and contact information of the representatives of the user association.
**Semi-structured interviews** to qualitatively understand stakeholder experiences with user associations and their perspectives on how user associations operate, their effectiveness, and enabling and hindering factors related to their functioning	Interviews (n=19) with members of users associations, government officials from national and sub-national governments, representatives of health insurance companies, pharmaceutical industry, patients associations and other members of civil society. Purposive sampling to select respondents across the following criteria: recognition by their peers and among the stakeholders in the health sector, representation of the different regions of the country, representation of the different entities, and public and private organisations of the health sector.	Interviews were conducted between September 2020 and March 2021on Zoom and ranged from 1.5 to 2.5 hours in duration. Each interview was led by a single member of the research team and other members of the team were also able to listen to the interview and pose clarifying questions or points for further elaboration. Interviews covered the following topics: (a) an introductory section to tease out characteristics of the informant and the institutions they represent, (b) questions on the informant’s involvement in the initiative, (c) questions on the functioning of the user associations and the enabling and limiting factors involved, (d) questions on the perspectives of the initiative for the future.
**Focus group discussions** to obtain rich details on the most salient factors identified via interviews	Focus groups (n=6) with 37 participants: members of insurer user associations (n=2), insurer citizen representatives and patient associations (n=2), members of the medical association (n=1) and people working in the pharmaceutical industry (n=1). Purposive sampling across the informant types and geographical variability.	Focus group were held on Zoom in June 2021 and ranged from 1.5 to 2 hours in duration. Each focus group was led by a facilitator (JB, MB-V) and other members of the team were able to participate. Focus groups covered the following topics: How the user associations function, categories of associations, major limiting factors and policy alternatives to improve the initiative.
**Policy discussions** to get feedback on preliminary results and recommendations, identify key factors not sufficiently considered and discuss the feasibility of and prospects for effectiveness of the policy recommendations	Sessions (n=6), with between two and six participants, one each for: representatives from government (Ministry of Health, local health authorities and oversight agencies), the Constitutional Court, associations of health insurance companies, insurance companies, leaders in the health sector and community leaders. Purposive sampling to cover the most relevant decision-makers in the health system or related to the protection of human rights in relation to healthcare access.	Discussions were held on Zoom in May and June 2021 and lasted for approximately 2 hours each. After presenting preliminary results and policy recommendations, the principal investigator (MB-V) opened the floor for participants and facilitated a discussion about the results (Do you agree with the results? are there any factors not sufficiently considered?) and the policy recommendation (do you agree with the recommendations?, are they feasible to implement?).

EPS, Entidades Promotoras de Salud.

10.1136/bmjgh-2022-009571.supp1 Supplementary data

### Data analysis

Quantitative data from insurance beneficiaries survey and secondary data sources were descriptively analysed, including calculating descriptive statistics and measures of association between indicators. We report point estimates and confidence intervals of key indicators for the study, including public awareness, use and participation in user associations. Further details of the results from quantitative data can be consulted in the online supplemental annex. Data from the health insurance beneficiaries survey were analysed in R using the {survey} package.^12–14^

Qualitative data were used primarily to explain the findings from the quantitative analysis. In particular, qualitative data collection aims to explain the functioning of user associations to understand why the initiative has progressed to the observed results (in terms of awareness, use, participation) and what are the key factors driving these findings. Interviews, focus group and policy discussions sessions were recorded and transcribed. Qualitative data were analysed thematically,^15^ beginning with deductive–inductive open coding of the data from in-depth interviews. For this we used the theoretical framework to define initial categories and codes to assign to the data and we let other codes emerge during the coding process. For the deductive process, we used a set of codes that underlined enabling and limiting factors for the user associations, classified using the process–content–context structure. These codes comprised information, motivation, cost, purpose, power-relationships, regulation, culture, politics, geography. Influential factors for the initiative that did not fit within any of the initial codes were added to the coding matrix. We then applied our coding framework to focus group discussion transcripts and policy discussion meeting notes. Afterwards, we identified relationships among categories and aggregated them to build an overarching understanding of how the user association initiative was designed (content), how it was actually functioning (process) and what are the most important reasons for the way the user associations function (context).

### Patient and public involvement statement

There were no patients involved in the study. Members of the public were involved as research participants, and their experiences, priorities and preferences shaped the policy recommendations arising from this research. While the intent of the research is to improve health insurance responsiveness to the general public, the public was not involved in setting the research questions or designing the study.

See also the reflexivity statement in the online supplemental appendix.

10.1136/bmjgh-2022-009571.supp2 Supplementary data

## Results

### Content: user associations as per the legal provisions

The origins of health insurance user associations can be traced back to Article 49 of the 1991 Constitution that states that the community will participate in the organisation of healthcare. This participation was further developed in the 1993 health reform that gives health insurance enrollees the right to join or create user associations (Art. 156, lit. h). These user associations would ‘represent them [insured people] before the EPSs [health insurance companies] and the health care providers’ (Art. 156, lit. h) and ‘strengthen the negotiating capacity, the protection of rights and community participation of the affiliates’ (Art. 157, par. 3). Moreover, in 1994, the national government mandated that health insurance companies guarantee community participation (Art. 9, Decreto 1757/1994) through user associations that ‘will look after the quality of services and defend users’ (Art. 10).

Although the 1993 law mandated that it was the government’s responsibility to ‘promote’ user associations (par. 3. Art. 157), the government later delegated this responsibility to the health insurance companies. In particular, health insurance companies have to ensure there is at least one user association in every department in which they operate, and they must ‘promote and strengthen’ participation in user associations by inviting the insured to create or join them (Circular externa 008, SNS). However, the law does not define how the government or health insurance companies are to promote the initiative. In particular, beyond the mandate to promote and support user associations, the law does not define specific mandates on education, resources or technical support that the government or insurance companies should provide.

Legal provisions have made it straightforward for the insured to form user associations: it requires as few as two affiliates signing creation minutes and submitting them to the *Superintendencia Nacional de Salud* (SNS, National Health Oversight Agency). Multiple user associations can be formed for any health insurance company (Art. 12, Decreto 1757/1994) and the insured can create an association even if they have not been ‘invited’ to do so by the health insurance company (Decreto 780/2016). It remains the users’ responsibility to create and operate the associations. The regulatory provisions do not endow user associations with financial support, but associations are allowed to charge membership fees (Art. 157, Par. 3°, Law 100/1993).

**Figure 1:**
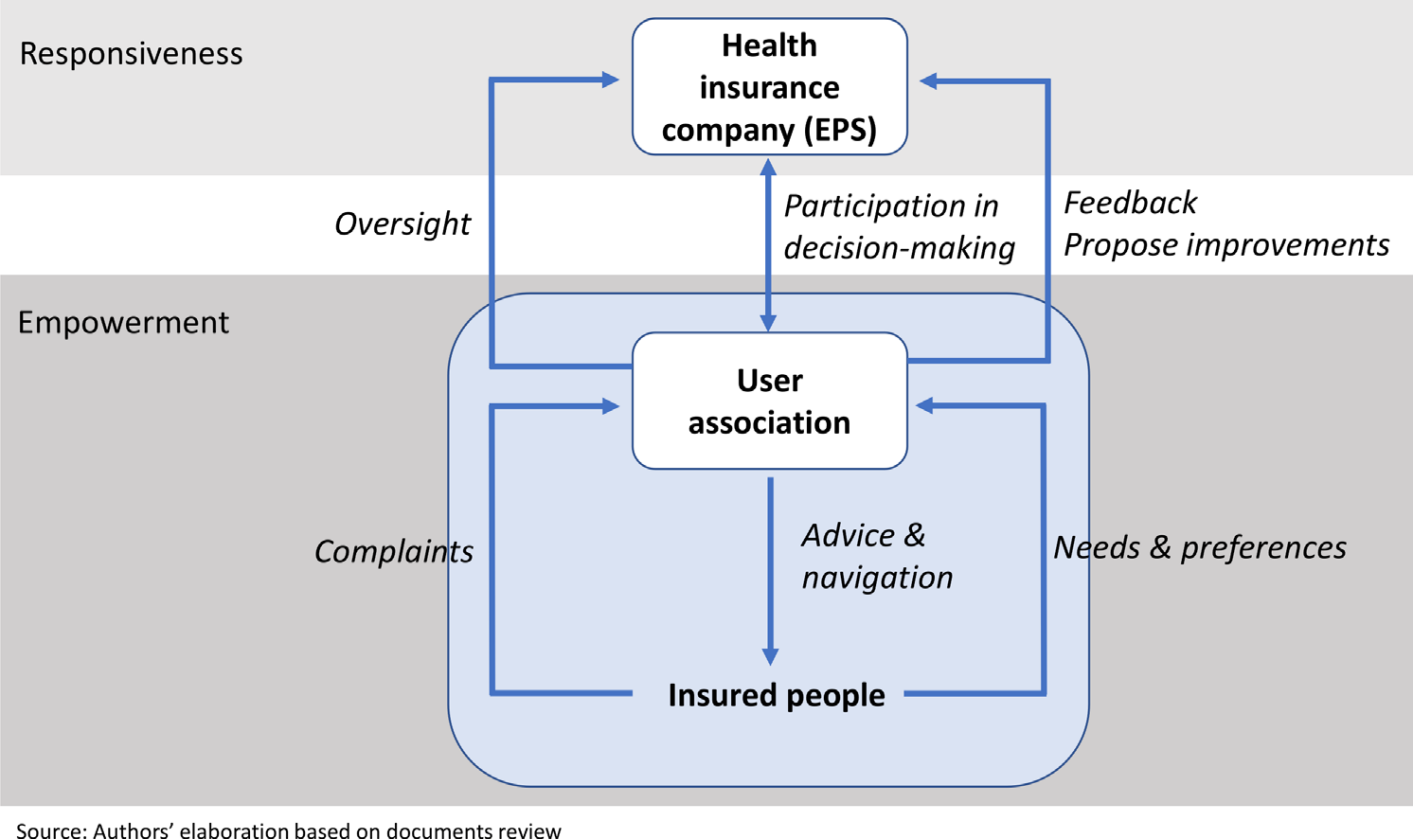
User association functions summarised. EPS, Entidades Promotoras de Salud.

User associations are supposed to provide advice to citizens on how to navigate the health system and access health insurance benefits, and solicit citizen needs, concerns and complaints relating to their health insurance company (Decreto 1757/1994), thereby empowering citizens exercise their insurance entitlements. The user associations are in turn supposed to share this feedback with the health insurance companies and suggest improvements, They are also envisioned as participants in decision-making within the health insurance companies. Through these functions, it was believed that the user associations would enhance the responsiveness of health insurance companies (figure 1).

Although health insurance companies are legally required to provide ‘adequate and timely processing of the concerns and requests of their enrollees’ (Circular externa 008, SNS), the user associations do not have any special means to enforce this. Thus, in the event of a breach by the health insurance company, the user association would need to use the regular complaint mechanisms of the health system (operated by SNS), as any other enrollee could.

The regulation dictates that there must be one seat for a user association representative on the board of directors of publicly-owned and mixed-ownership insurance companies (Decreto 1757/1994). After a legal battle led by user association representatives, a 2012 ruling from the Constitutional Court enforced that and mandated the EPSs to comply with the regulation.^16^ Yet, the regulation does not define the details of how the EPSs should adhere to user association input.

### Process: how user associations actually function, and their effect on the health insurance scheme

Health insurance affiliates have created at least 1289 user associations for the 40 different health insurance companies currently operating across the 32 departments of Colombia.^17^ There is compliance with the regulation that there must be at least one user association in each department where a health insurance company operates. For 23 EPSs in 27 different departments, there is more than one user association (from 2 to 116 user associations) in a single department. While there is no requirement that user associations be formed in each municipality, there is at least one user association in 69% of the municipalities in the country. This is no minor achievement because there are only a handful of social interventions that reach so many municipalities. Yet, that means that there are 348 municipalities out of the 1117 (31%) without any user association. User association density—defined as the percentage of insurance beneficiaries that have a user association in their EPS and municipality—ends to be lowest in the south-east of the country (towards Orinoquía-Amazonía), which also has a lower population density, is marked by poorer health system performance, poorer access to public services, lower household incomes and weaker governance (see online supplemental figure A for a geographical density map).

10.1136/bmjgh-2022-009571.supp3 Supplementary data

**Figure 2:**
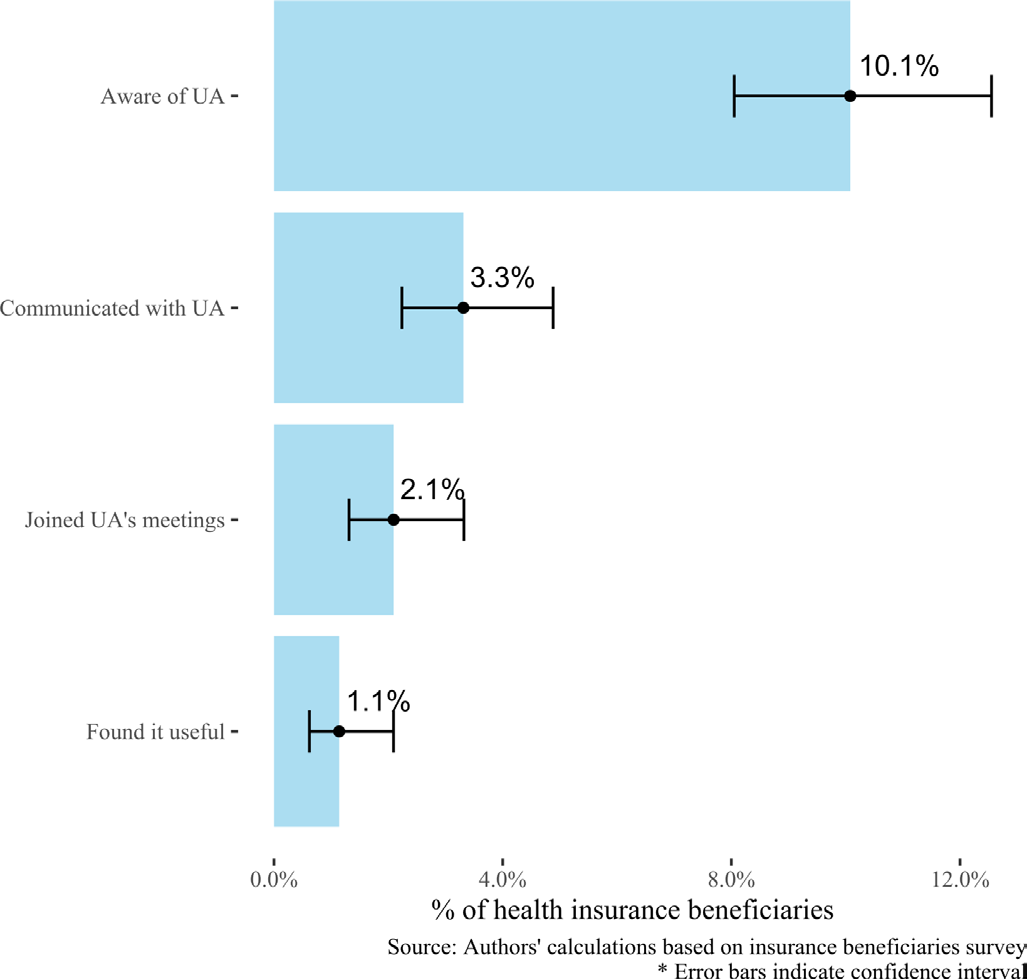
Funnel of participation in user associations.

Despite the high number of user associations, the health insurance beneficiaries survey shows that just a small fraction of the population is aware of and engages with user associations (figure 2). Overall, only 10% of people with health insurance know of any user associations, just 3% have had any communication with a user association, only 2% have joined at least one user association meeting and less than 1% reported finding them useful.

Knowledge about and engagement with user associations was not significantly different between users in the contributory and subsidised schemes. Awareness of user associations was significantly higher among those who used their health insurance benefits (11% vs 4%, p<0.05) and those who had a medical visit (15% vs 2%, p<0.05) in the last year versus those who had not, and those who had a chronic disease or disability versus those who did not (15% vs 8%, p<0.05).

User association members and patients interviewed reported insufficient promotion of user associations by governments (both national and local) and health insurance companies. While some people reported receiving letters informing them about user associations, most reported that health insurance companies limited their publicisation efforts to a single informational page on their websites. Not only does this require active searching, but it also excludes the 30% of the population who lack routine access to the Internet.^18^

The EPS [health insurance company], what does it contribute to inviting participation? […] A little web page for everything about user associations. It’s well presented but not everyone goes online to dig, sometimes the user doesn't even know [that user associations exist]. (Interview 46, User associations and civil society)

I think that only those who are involved in these user associations know the potential of this participation mechanism, but the rest of the people do not. […] The vast majority of EPS users are unaware of this participation mechanism. (Interview 12, National Government)

The number of people participating in user associations is usually small. Of the 27 respondents in the user association member survey (likely a sample of highly engaged members with easy access to Internet), 7 reported that their user association was composed of a single active member and none of them reported more than 10 active members.

User associations leaders work on a voluntary basis and had to pay for their own transportation, communications, meeting venue and any other costs, including costs associated with reaching out to the insured. A few health insurance companies were reported to provide some resources (eg, an email address, a venue for meetings), but support was limited and up to the discretion of the specific company.

Training for user association members was irregular and non-standardised. While some user associations received training or strategy development support from the SNS or health insurance companies, others received nothing. Respondents from user associations, government and civil society agreed that members’ lack of knowledge or technical skills was an issue, including on the complexities of the healthcare system and health insurance financing.

The volunteer (unpaid) nature of the role, lack of financial support for activities and limited training has made membership in user associations primarily feasible to retirees and individuals seeking to solve specific issues that they or their family members are facing with healthcare; among the 27 members who completed our survey, 20 reported participating to try to solve a personal issue, most were over 65 years in age, and none had full time employment.

Despite some exceptions and success stories, overall, user associations were reported to have minimal influence on health insurance companies and played a marginal role in supporting citizens in accessing and navigating the health insurance system. Some user associations were entirely non-functional, and were reported as having been created to meet the regulatory requirement without any genuine membership.

There are some that are created simply to comply with the norm that requires them to have an association, but it is an association that is constituted and is never seen again. (FG315, User associations and civil society)

User association members reported limited outreach to help other insurance participants/enrollees navigate the health system or to solicit patient complaints or suggestions. Even the most active user associations reported that they could reach (eg, provide information, receive a complaint) hundreds to a few thousand users in a year. This is a lot given the limited resources they have, but little in relation to the overall population. Most user associations were said to be limited to working as ‘complaints processors’ (Interview 33, EPS representative) wherein they passed on individual requests and complaints to the insurance companies or the SNS when approached by someone with a specific issue.

Among associations whose members attempted to take on further activities, respondents explained that health insurance companies at times actively resisted user association involvement in decision-making and were free to disregard user association suggestions and recommendations. User associations lacked mechanisms to compel health insurance companies to seriously consider their concerns and respond accordingly. User associations’ lack of influence and power over health insurance companies was discussed as a fundamental problem.

We do not have the right to influence what they [the EPS] consider important. They are clearly limited to receiving requests, but these are not going to be a strong reason for them to make a decision. (Interview 45, User associations and civil society)

User associations have the right to ‘democratically elect’ one representative to participate in the EPS Board of Directors, for public or mixed EPSs. Being part of the board of directors should help compelling the EPS to respond to the associations’ feedback and not only respond to their shareholders, who typically have a seat on the board. Yet, interviewees reported that EPSs tried to impede such participation altogether or circumvented it, by managing to place on the board a representative from a friendly user association who would do little to try and convey user’s needs and expectations to the board.

Although interviewees characterise most user associations as non-functional, complaints-processors, struggling for resources or contending against a reluctant EPS, we identified success stories where there was a supportive relationship between the EPS and the user association. In one of these cases, interviewees reported a close collaboration between the EPS and the user association, who were said to have ‘an open door, a direct line, and a permanent interaction’ (Interview 33, EPS employee) with regional health insurance managers. User association members took part in the microlevel decision-making of some committees, but not in the Board of Directors, where more strategic high-level decisions were taken. A member of this user association reflected that they gained this influence because the EPS wanted to learn about and rectify user complaints, in order to retain users.

The user association [is very important] for the operation of the EPS. Why? Because through the user association, they can collect all the disagreements, the complaints […]. And that’s beneficial for them because in the event that there is a user or many users who relocate [switch to another insurance company] due to bad service provision—oh mamita!—there once and for all, rest assured that the financial part of the EPS goes down because that talk goes everywhere (interview 46, User associations and civil society).

The key driver of the success story was ultimately the EPS’s willingness to support and collaborate with the user association. Without such a willingness, even empowered leaders end up struggling to make their voices heard due to lack of resources. But the factors leading to a favourable stance of the EPSs towards user associations may be complex. Sometimes even EPS that may find some value in user associations, may not be willing to support them due to a combination of factors that include the cost of doing so, perceived legal barriers to do it and other idiosyncratic factors such as who is leading the EPS or the history of the insurance company. For example, in the case discussed above, the Chief Executive Officer of the insurance company was reported to truly believe in community participation and therefore, designated a top-ranking official within the EPS to lead community participation issues and assigned her a budget. Other success stories were reported in EPSs with roots in the community (mutuales) and among EPSs that took part in one of the EPS guilds in the subsidised scheme, which also engages in some efforts to promote community involvement in health insurance.

### Context: factors affecting the functioning of user associations

The functionality of user associations is grounded in the Colombian health system context, broader perspectives on participation in Colombian society, and the Colombian legal and judicial context. While the first two contextual features hinder user association effectiveness, the legal and judicial context is a potentially significant enabler.

In the Colombian health system model, health insurance companies have an incentive to contain costs while consumers are expected to demand quality through market competition. Interviewees reported that EPSs may fear that an empowered user association could lead to increased health expenditure through increased demand or by requesting improvements in services.

I think that the last thing that the EPS wants to see [in the user associations] is the involvement of people who have made ourselves known […] Because they know [we can guide the] new patients [to get the services they need]. I really feel that they put up a great barrier so that we [activists] […] cannot be leaders in the user association. (Informant 35, Focus Group 3, User associations and civil society)

The user association tries to be a bridge between the [health insurance] administration and the community, and fight for better user services, but it becomes very difficult when the administration doesn’t want this. (Informant 311, Focus Group 3, User associations and civil society)

Theoretically citizens can switch health insurance companies when dissatisfied with their services. In reality however this regulatory mechanism falls short. The insured may feel that all EPSs offer similar services, may find the bureaucratic requirements to switch burdensome, may lack the technical knowledge necessary to assess quality or may live in a geographical area without another EPS option. Thus, health insurance companies support the creation of user associations in order to comply with regulations but have little incentive to empower user associations to take on a meaningful grievance redressal or oversight function.

In terms of the broader social context, the population has low awareness of the existence of user associations and low motivation to engage in participatory activities. Low motivation is grounded partially in the design features of the user association (eg, lack of resources to support user association activities and a lack of influence over EPSs as discussed above) and in broader contextual features of Colombian society. In Colombia, people see a risk of violence against social leaders and there is, to some extent, a lack of belief in participatory and democratic instruments.

Unfortunately this violence, …, so many community leaders murdered, that people do not want to participate in these processes anymore; fear has invaded us (Interview 21, Sub-national government).

I think that Colombia has in general, like the countries of Latin America, […] an apathy of citizen participation and I think that we are in a context of apathy for democratic instruments. (Interview 41, User associations and civil society)

In terms of the legal and judicial context, there is a human rights approach underlying the user association initiative that could facilitate future improvements. The right to participate is a key part of the right to health, which makes the initiative enforceable through the courts, using expedited rights protection mechanism called ‘tutela*’*. A tutela is a lawsuit that can be filed without the need for a lawyer or any legal assistance and must be resolved in 10 days. The population is generally aware of this mechanism and its effectiveness. It is highly regarded and typically portrayed as one of the most important social achievements of the 1991 Constitution. This context has enabled user associations for public EPSs to demand a position on the board of directors. Although this positive ruling has so far proven insufficient to overcome the numerous other barriers facing user associations, future lawsuits may strengthen user association power and support—for example, they could try to get representation in the decision-making process of private insurance companies.

## Discussion

Colombia’s user associations have an expansive mandate to represent the interests of the insured in negotiation with health insurance companies, enable community participation in health insurance company decision-making, ‘defend users’, and oversee the quality of health services. However, this study found that user associations generally do not function as vehicles for accountability and citizen participation in the health system. While they have been formed in each department across the country, few people know about their existence and membership are generally limited to just one or two people, many of whom joined or formed an association to solve a specific personal issue they were facing with their healthcare. User associations lack access to financial resources, such as an adequate budget to support outreach communication, and are not provided with systematic training on the complexities of the health insurance system. Most fundamentally, private health insurance companies are not compelled to involve user association members in decision-making and are not generally motivated to seek their assistance in soliciting patient complaints and devising solutions. User associations lack access to any additional or special channels to influence health insurance companies. Although public and mixed-ownership EPSs have been mandated to include a user association member on their board of directors, this has not translated in genuine community involvement in decision-making.

We present a typology of user association activity and summarise the causes of varying levels of activity and functionality (table 2). The typology results from the analysis of the different interviews, focus groups and policy consultation meetings. We grouped together the interviewee’s descriptions of specific user associations that shared similar traits, for example, how those associations operate and the reasons that may have led to such a result. We then move beyond our findings (as presented using the content–process–context framework) and discuss them in view of broader theories of community participation and in doing so wearranged the groups using the conceptual heuristics of Arnstein’s ladder of citizen participation^19^ and finally we assigned descriptive names to each type of user association.

**Table 2:** Typology of Colombia’s health insurance user associations

Type of user association	Definition	Causes
No user association	Municipalities without any user association formed *About 1/3 of all municipalities lack a user association*	There is no legal mandate that EPSs have to form user associations in each municipality. Few people know that user associations can be formed, due to insufficient promotion of the initiative by EPS. Low citizen motivation to form them. Geographical and infrastructure challenges make it difficult to physically meet.
Coopted user associations	User associations wherein some or all members are closely aligned with the EPS. *Most associations created for the purpose to comply with the regulation may be coopted*	EPSs see no value in an active and independent user association. The EPSs seeks to comply with regulatory mandates (make sure there is at least one association where they operate), while avoiding independent user participation in decision making. EPSs seek to avoid pressure to change their operation to accommodate user needs and expectations. Users unaware of cooption or unable/unwilling/afraid to try to regain citizen control.
Transient	User associations that exist solely to solve a specific problem faced by a member. *A sizeable minority of user associations could be classified as transient*	Operating a user association is not financially rewarding and user association members receive little support. Joining or forming a user association when seeking to resolve a specific issue however, can be rapidly gratifying and rewarding. EPSs may be far more willing to work with a user association to solve one specific problem (and then see the association return to dormancy) than to support an ongoing and broadly active user association.
Complaints-forwarder	A user association that exists over the long term but that engages primarily in passing grievances from citizens to the EPS. This type of user association does no active outreach to the community to identify grievances, nor does it demand change from the EPS as a result of grievances. *Many active associations may be complaints forwarders*	User association members are not strongly motivated, lack skills and lack support The EPS is satisfied with this sub-optimal operation of the user association because they gain information about enrollee satisfaction but are not forced to resolve all issues identified
Struggling but contending	User association attempts to educate, engage and solicit grievances from enrollees, and attempts to demand change from the EPS; however, they achieve very marginal results *A few user associations could be classified as struggling but contending*	Members and leaders are motivated to solve broad community issues. Members lack resources (eg, transport, communication, venue). The EPS is hostile to user association demands that might potentially result in higher healthcare or administrative costs for the EPS to bear. User association may also be competing with another user association that has been co-opted, making it even harder to be able to participate in decision-making bodies within the EPS.
Empowered and effective	Motivated members with access to sufficient operational resources that interacts with a receptive EPS to improve the quality of healthcare and access to comprehensive insurance coverage. *Very few user associations appear to be empowered and effective*	The EPS has created an enabling environment by providing resources for the user association and by welcoming the user association’s input on the company’s operations. The EPS leadership values user associations as a means to improving quality of care provided to better retain enrollees. User association members are particularly competent and motivated to improve the health system (perhaps due to prior engagement in activism).

EPS, Entidades Promotoras de Salud.

Despite the current challenges facing user associations, almost all interviewees believed there is potential in the initiative and considered it worth trying to improve their functioning. The literature, unfortunately, does not provide many additional insights on how to improve user associations because there do not seem to be equivalent experiences elsewhere. Although there are important experiences on community involvement in health insurance systems, after systematically searching the literature, we could not find an initiative like the user associations, that aims for an organic community involvement to operate at the departmental level for every insurance company in a multiple-payer system that competes for enrollees.

Yet, the range in functionality illustrated in the typology—with most associations concentrated at the absent and co-opted end of the spectrum—echoes findings from attempts in other settings to institutionalise community participation in health through committees. Health committees have often been stymied by lack of clarity about their role, mandate and authority,^20^ lack of training and support^21–23^ as well as insufficient discretionary power to effect change in the health system.^24–27^ A lack of awareness about the existence of committees and community scepticism about their utility undermined health committee participation, influence and value in many settings.^21 28–30^

Participatory structures for health system governance have moved along the spectrum towards empowerment and effectiveness when many of these issues are resolved, usually with strong and institutionalised political support. In India, for example, health committees were activated through widespread community mobilisation, training and capacity building inputs for members, and access to some budgetary and political power.^31 32^

These experiences in different contexts exemplify similar challenges as the ones faced by health insurance user associations in Colombia. And they also illustrate that overcoming barriers to participation may be possible with strong support. To move towards empowered and effective user associations, Colombia’s Ministry of Health should develop and apply policy measures to increase public awareness of user associations, provide members with adequate resource support, and endow user associations with clear legal rights to participate in decision-making bodies of health insurance companies towards increasing health insurance responsiveness (table 3). These policies should be developed in a consultative manner under the Ministry of Health’s leadership, involving EPS managers, user associations, health activists, healthcare providers and citizens.

**Table 3:** Recommendations for building empowered and effectual user associations

Goal	Recommendations to achieve this goal
Ensure widespread public awareness of user associations and their role	Strengthen the promotion of user associations by government agencies and define in detail what each EPS must do to ensure every insurance enrollee knows that user associations exist, what can they be useful for and how they can participate if they want. The clearly defined responsibilities should be non-delegable. Discuss and agree with the stakeholders the level of operation for the user associations, currently regulated at the department level in each EPS. Yet, interviewees indicated that an optimal operation of the user associations require proximity to the insurance beneficiaries and therefore, a more granular level of operation (eg, municipal level, or even lower as suggested by at least two interviewees) could help user associations to be more effective.
Provide adequate resource support for user associations	User associations require clearly defined sources and amounts of funding and in-kind resources to support their work (a room, email address, funds for transport and communications, training) User associations require routine training, which is currently provided by the SNS, but that interviewees report as insufficient.
Genuine decision-making power	Clearly define the user association’s role in terms of whether they have a voice and vote, and ensure their ability to participate in all types of meetings of the health insurance board. Also provide user associations with a seat on the board of directors of private EPSs. Mandate EPSs to involve user associations in lower-level decision-making bodies (eg, local committees in the EPS that make decisions about procurement or deal with local networks of providers). The decisions made at these lower-levels are probably closer and more relevant to the users, and in addition, there might be less resistance from the EPSs to allow participation in such instances than it would in the board of directors where more strategic macro-decisions are made.

EPS, Entidades Promotoras de Salud; SNS, Superintendencia Nacional de Salud.

Overall, while Colombia provides a strong regulatory and legal environment in support of citizen participation generally and health insurance user associations specifically, these facilitators have not been sufficient to overcome the substantial operational and contextual barriers to citizen empowerment and health system responsiveness. Knowledge of, and participation in, user associations is limited. User associations tend to exist as passive or wholly inactive bodies—and have at times been co-opted—due to a combination of challenges at the level of content (voluntary, *ad-honorem* work for a potentially time-intensive activity, few tools to trigger responses or actions from the insurance company), process (weak promotion of the initiative, EPSs that at times may try to obstruct *user associations*’ work) and context (low competition between health insurance companies as well as a hostile environment to participation on account of violence against social leaders). This has led to a situation where the user association initiative suffers from low awareness among the population and low participation levels that can hardly lead to empowered enrollees and a more responsive health insurance. Yet, most stakeholders value the space to participate and still see potential in the initiative. This warrants effective changes in policy to strengthen user associations such that they are provided this needed participation space and bring about long term, effective citizen empowerment that was envisioned at the time of their establishment.

## Conclusion

Health insurance user associations in Colombia, as they currently function, are not an effective vehicle for citizen participation in the health system. They cannot have a widespread impact in the system because they do not reach a large fraction of the insured people. User associations can sometimes serve to empower a few insurance beneficiaries. But even empowered users frequently face unsurmountable barriers to make their voices heard. Therefore, user associations generally fail to trigger a positive system response. But in the midst of all these difficulties, there are motivated leaders who do valuable work and achieve important results, despite structural barriers. Policy changes to eliminate those structural barriers are warranted.

### Limitations

A number of limitations of this study arise from the fact that it was undertaken during the COVID-19 pandemic and the governments’ response with general lockdown and social distancing measures. This forced us to change the primary data collection approach. In particular, we could not do face-to-face interviews as originally planned. Instead, we had to rely on telephone interviews for the insurance beneficiaries survey and online meetings via Zoom for the in-depth interviews and focus groups.

As a result of the lack of reliable contact information for all user association members, the survey for them uses a non-random sample. Therefore, one cannot generalise those results to the whole population of user association members and thus, we use those results rather qualitatively. Yet, it is likely that those that responded the survey are some of the most active user association members.

### Further research

This study identified several challenges that health insurance user associations face and also outlined a set of policy measures to strengthen user associations. This leaves interesting questions for future research, such as, how to best sensibilise health insurers to support and take advantage of community participation. In which decision-making bodies are user associations more useful. How to involve a broader population in the user associations. In addition, even though they were not part of the study, during the data collection we kept receiving information of other types of associations that operate in the health system. In particular, patients associations seem to be a powerful force in the health system and apparently can be frequently more effective than health insurance user associations. Key factors such as motivation to participate are different, but it would be worth exploring in more detail those other kind of citizens participation in the health sector to see what the health insurance user associations can learn from those other experiences.

## Data Availability

Data are available upon reasonable request. Data that can be anonymised are available upon request.
