# Supplementary material for: How can community participation strengthen a health insurance system? The case of health insurer’s user associations in Colombia
Source: BMJ Glob Health. 2022 Sep 16;7(Suppl 6):e009571. doi: 10.1136/bmjgh-2022-009571 (PMC9486220; doi:10.1136/bmjgh-2022-009571)
Supplement: Supplementary data [file bmjgh-2022-009571supp001.pdf]

Knowledge and Use of Participation Mechanisms

		Total		Contributory		Subsidized		t-test
	N	%	SE	%	SE	%	SE	p-value
**User Association**								
Knows any user association	1311	10.1	0.005	11.7	0.008	8.4	0.007	
Has participated in any user association	1311	2.1	0.003	1.5	0.003	2.7	0.004	

Note: * p-value <0.1, ** p-value < 0.05, *** p-value <0.01

Sample Characteristics

	INSURANCE REGIME		TOTAL
	Contributory	Subsidized (Sisben)	
	%	%	%
**Age**			
18 to 25	14	11	13
26 to 40	27	29	28
41 to 55	23	26	25
56 to 65	19	19	19
66 to 88	17	14	15
N/R	0	1	0
**Sex**			
Male	47	49	48
Female	53	51	52
**Education**			
None	1	5	3
Primary incomplete	9	20	14
Primary complete	6	12	9
Secondary incomplete	7	12	9
Secondary complete	16	24	20
Technical incomplete	3	5	4
Technical complete	15	8	12
University incomplete	7	5	6
University complete (Professional)	22	7	14
Specialization, Masters or Doctorate	15	2	9
N/R	1	1	1
**Region**			
Caribbean	13	31	22
Antioquia + Eje Cafetero	24	15	19
Bogotá	29	6	18
Pacific	13	19	16
Central	12	15	14
East	9	14	11

**Socioeconomic Strata**			
Strata 1	19	56	37
Strata 2	29	35	31
Strata 3	33	8	21
Strata 4	12	1	7
Strata 5	5	0	3
Strata 6	2	0	1
**Has communication tools:**			
Email	73	47	60
Text message	81	71	77
Smartphone	77	60	68
Computer	61	23	42
Internet Access	82	47	65
**Time enrolled in current EPS**			
Less than 6 months	8	5	6
6 months to 1 year	7	9	8
>1 year to 2 years	13	10	12
>2 years to 5 years	19	20	20
>5 years	53	56	54
**Last time used EPS services**			
Less than 6 months	61	50	56
6 months to 1 year	22	27	25
>1 year to 2 years	7	12	9
>2 years to 5 years	4	7	5
>5 years	3	2	2
Never	3	2	3
**Diagnosed with NCD**	29	17	23
Total Interviewed	666	645	1,311
Population represented	16,795,014	15,723,832	32,518,930
%	52	48	100
