# Supplementary material for: How can community participation strengthen a health insurance system? The case of health insurer’s user associations in Colombia
Source: BMJ Glob Health. 2022 Sep 16;7(Suppl 6):e009571. doi: 10.1136/bmjgh-2022-009571 (PMC9486220; doi:10.1136/bmjgh-2022-009571)
Supplement: Supplementary data [file bmjgh-2022-009571supp002.pdf]

**Appendix S1 – Reflexivity Statement**

**1. How does this study address local research and policy priorities?**

This study aligns with local policy priorities by addressing social participation in health, which has been gaining importance in recent years in Colombia. For example, in 2017 the Ministry of Health adopted the "Policy of social participation in health " that aims to "Define and develop the guidelines that allow the State to guarantee the right to social participation in health and its strengthening and to citizens the appropriation of mechanisms and conditions to exercise social participation in health in decisions for the fulfilment of the fundamental right to health within the framework of the Statutory Health Law and in harmony with the Comprehensive Health Care Policy - PAIS". This policy reveals a renewed interest in social participation in the health sector that puts this topic on the front of the policy agenda.

This renewed policy relevance warrants a research agenda on social participation in the health sector; a topic that has been relatively neglected in the country for many years. Thus, this study also addresses local research priorities by contributing to fill in knowledge gaps on how social participation can be beneficial for the health sector. The priority to understand social participation in the health sector has been formalized in the research priorities jointly outlined by the Ministry of Science Technology and Innovation and the Ministry of Health, that include includes science, technology and innovation to strengthen the social appropriation of knowledge through communication, exchange and citizen participation programs for the health sector among another prioritized sector.

**2. How were local researchers involved in study design?**

This research was designed and led by a research team based in Colombia. It was the team in Colombia that decided to answer the call from the Alliance for Health Policy and Systems Research. These investigators had the most experience conducting, directing, or organizing national research collaborations (MBV, JB). The lead investigators worked closely with other Colombia based researchers who had different strengths necessary to pool the efforts and knowledge required for the development of the research. These strengths were: a) knowledge of the dynamics, leadership, operation, and participation in the different initiatives under study in health in the country (MH); b) management of secondary information sources for the study and experience in database management (HR); and c) the skills for the development of qualitative methodologies such as conducting interviews and focus groups (JB).

**3. How has funding been used to support the local research team?**

This project provided funding (US$ 75,000) used primarily for the local researchers and to collect primary data.

**4. How are research staff who conducted data collection acknowledged?**

Data collection was conducted by the researchers (see specific roles in the contributions section of the manuscript) and all of them are authors. Data collected for the survey of insurance beneficiaries was contracted out with a local firm specialized in surveys and data collection and they are acknowledged in the manuscript.

**5. Do all members of the research partnership have access to study data?**

All members of the partnership have access to data.

6. **How was data used to develop analytical skills within the partnership?**

The researchers analysed the data according to their qualitative or quantitative strengths and abilities. The teams are then paired up to triangulate information and leverage multidisciplinary skills in order to develop a more comprehensive analysis of the investigation.

7. **How have research partners collaborated in interpreting study data?**

All authors contributed to interpreting study data. There was a first interpretation of study data carried out by the authors leading the study. Afterwards, through exchanging technical reports, feedback and during the writing process, all authors collaborated in the interpretation of the data and study results.

8. **How were research partners supported to develop writing skills?**

The research team writing this statement is predominantly composed of high-level academics with a native Spanish language background. Therefore, the support of an international senior researcher in the publication of this type of article was important.

9. **How will research products be shared to address local needs?**

We have developed a post-publication dissemination plan to distribute study findings and policy recommendations to the general public in Colombia. In addition, we reached to newly appointed health authorities to communicate the results of the study to the new government that will soon take office. Finally, this study will be presented in the 7th Global Symposium on Health Systems Research (HSR2022) which will take place in Bogotá, Colombia. This broadens the audience to an international community of health systems researchers, policymakers and practitioners from around the world and at the same time will be a good opportunity to discuss the policy implications with the decision makers, considering that a representative of the Ministry of Health has been invited.

10. **How is the leadership, contribution and ownership of this work by LMIC researchers recognised within the authorship?**

The manuscript is led by Colombia based authors and they are the first authors. Authors MBV, EAS, and JB worked as part of the core authorship team on the conception and development of the manuscript. The other authors MVU, MH, and HR acknowledge their participation in preparing the data collection tools and, in the analysis, as did the main team. From what we recognize that the authoring team is predominantly in middle-income countries. The authors in high-income countries comprise IM who made substantial contributions to the study design, analysis and interpretation of results and KS contributed to the critical revision of drafts.

11. **How have early career researchers across the partnership been included within the authorship team?**

We acknowledge we did not include early career researchers in the partnership.

12. **How has gender balance been addressed within the authorship?**

Seven authors are female (MBV, JB, IM, MVU, MH, HR, KS) and one author is male (EAS).

**13. How has the project contributed to training of LMIC researchers?**

The authorship team is primarily composed of senior researchers. All the authors based in low- and middle-income countries are especially senior researchers. Research funding leveraged as part of this project will support employment of a junior researcher based in Malawi.

**14. How has the project contributed to improvements in local infrastructure?**

This project has not directly contributed to improvements in local infrastructure.

**15. What safeguarding procedures were used to protect local study participants and researchers?**

The study was conducted during the Covid-19 pandemic. As a safeguarding procedure of researchers and study participants, all interviews and focus groups were conducted using non-face-to-face procedures (Zoom meetings, telephone calls).
