# Supplementary figures and images for: How can community participation strengthen a health insurance system? The case of health insurer’s user associations in Colombia
Source: BMJ Glob Health. 2022 Sep 16;7(Suppl 6):e009571. doi: 10.1136/bmjgh-2022-009571 (PMC9486220; doi:10.1136/bmjgh-2022-009571)

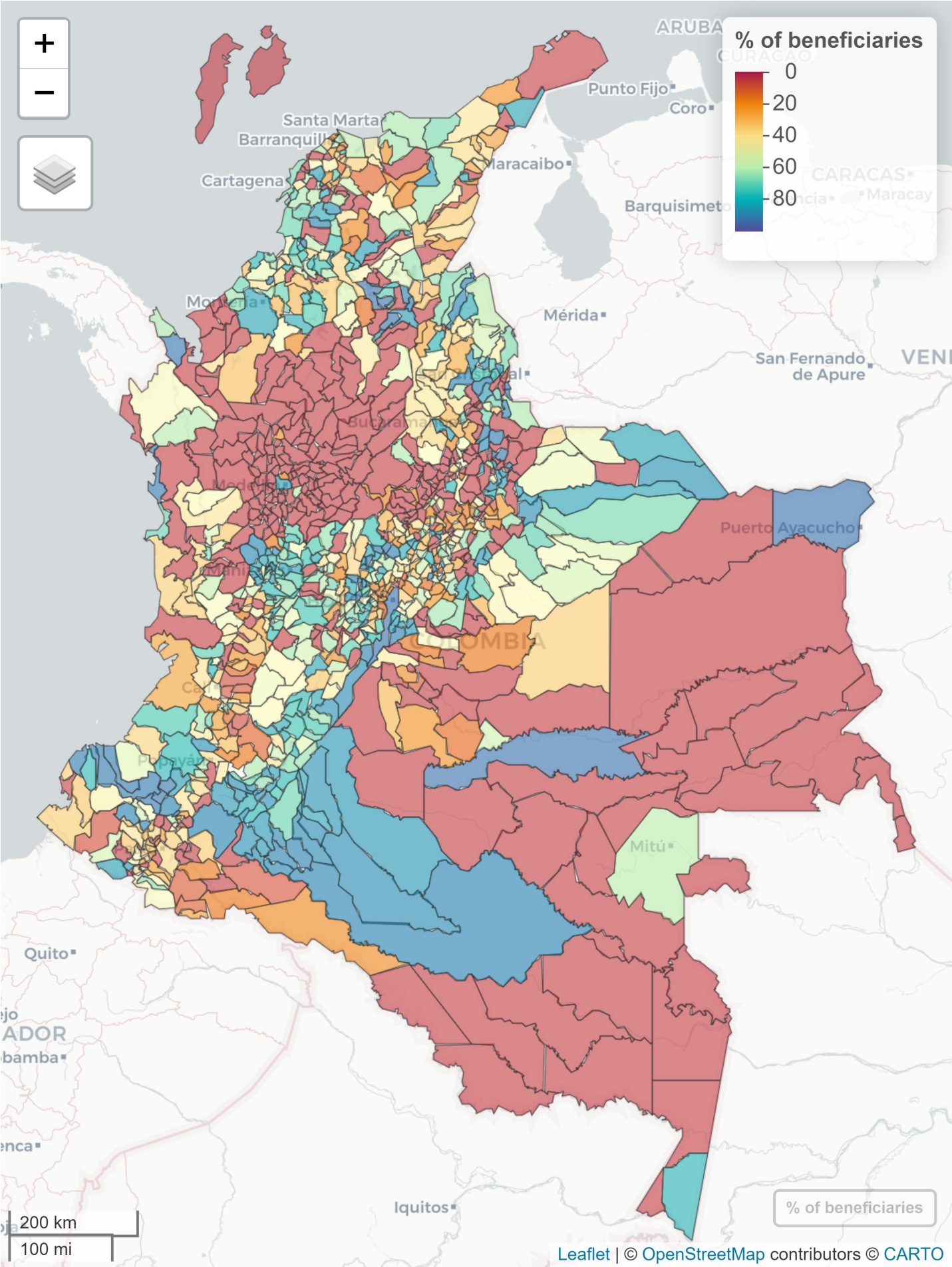

Supplement: Supplementary data [file bmjgh-2022-009571supp003.pdf]
